# Coarse particulate organic matter dynamics in ephemeral tributaries of a Central Appalachian stream network

**DOI:** 10.1002/ecs2.2654

**Published:** 2019-03-18

**Authors:** KEN M. FRITZ, GREGORY J. POND, BRENT R. JOHNSON, CHRIS D. BARTON

**Affiliations:** 1 Office of Research and Development, National Exposure Research Laboratory, U.S. Environmental Protection Agency, Cincinnati, Ohio 45268 USA; 2 Office of Monitoring and Assessment, U.S. Environmental Protection Agency, Region III, Wheeling, West Virginia 26003 USA; 3 Department of Forestry and Natural Resources, University of Kentucky, Lexington, Kentucky 40546 USA

**Keywords:** connectivity, deposition, ephemeral tributary, lag function, leaf litter, seasonality, storage, transport, wood

## Abstract

Headwater ephemeral tributaries are interfaces between uplands and downstream waters. Terrestrial coarse particulate organic matter (CPOM) is important in fueling aquatic ecosystems; however, the extent to which ephemeral tributaries are functionally connected to downstream waters through fluvial transport of CPOM has been little studied. Hydrology and deposition of leaf and wood, and surrogate transport (*Ginkgo biloba* leaves and wood dowels) were measured over month-long intervals through the winter and spring seasons (6 months) in 10 ephemeral tributaries (1.3–5.4 ha) in eastern Kentucky. Leaf deposition and surrogate transport varied over time, reflecting the seasonality of litterfall and runoff. Leaf deposition was higher in December than February and May but did not differ from January, March, and April. Mean percent of surrogate leaf transport from the ephemeral tributaries was highest in April (3.6% per day) and lowest in February (2.5%) and May (2%). Wood deposition and transport had similar patterns. No CPOM measures were related to flow frequency. Ephemeral tributaries were estimated to annually contribute 110.6 kg AFDM·km^−1^·yr^−1^ of leaves to the downstream mainstem. Ephemeral tributaries are functionally connected to downstream waters through CPOM storage and subsequent release that is timed when CPOM is often limited in downstream waters.

## INTRODUCTION

Although landscapes are comprised of freshwater and terrestrial ecosystems, wet and dry ecosystems are largely studied in isolation (Soininen et al. 2015). However, there is growing recognition of the vital importance of cross-ecosystem fluxes of energy and materials between wet and dry ecosystems for their sustainable management (Anderson et al. 2008, Bartels et al. 2012). A key exchange across the land–water interface that affects community structure and ecosystem function in temperate forested streams is the seasonal input of terrestrial coarse particulate organic matter (CPOM) as deciduous leaves and wood (Fisher and Likens 1973, Richardson 1991, Wallace et al. 1999).

Because of the hierarchical, branching structure of river networks, headwater tributaries typically represent much of the channel length in river networks (Horton 1945, Leopold et al. 1964). Therefore, headwater tributaries are a dominant interface for the seasonal organic matter flux from upland forests to downstream waters, such as rivers, floodplains, lakes, and coastal waters. Headwater tributaries can have year-round or perennial flow, but across different regions, many headwater tributaries cease flowing for part of the year (Costigan et al. 2017). While there is a growing interest in the study of temporary tributaries (Acuña et al. 2014, Arthington et al. 2014, Datryet al. 2016), much of the research has focused on tributaries that have intermittent or seasonally continuous flow lasting longer than a month. Despite the widespread abundance of ephemeral tributaries—those that flow in direct response to precipitation and snowmelt—their connections to downstream waters have been less studied than their intermittent and seasonally flowing counterparts (Boulton 2015). This pattern also applies to CPOM dynamics within temporary tributaries (reviewed by von Schiller et al. 2017) where ephemeral tributaries have been less studied (but see Jacobson et al. 2000, Hutmacher et al. 2015). Studying how habitats, like ephemeral tributaries, that alternate from dry to wet function as ecosystem interfaces will foster research that spans the historical boundaries separating terrestrial and aquatic research (Soininen et al. 2015).

Connectivity has been characterized according to the functions by which tributaries or wetlands affect the fluxes of materials, organisms, and energy to downstream waters (Leibowitz et al. 2018). According to the functional classification framework, tributaries and wetlands can be functionally connected to downstream waters based on net differences in terms of the quantity, form, and timing of material or energy fluxes over a given unit of time (Leibowitz et al. 2018). Tributaries or wetlands may be functionally connected as a source or a sink to a downstream water where there is a net increase or net decrease, respectively, in a material or energy source by processes occurring within the tributary or wetland before that material or energy is subsequently transported to downstream waters. Processes that do not change the quantity of a material or an energy source but change the form and timing of fluxes (through processing and storage) to downstream waters are functionally connected through transformation and lag, respectively.

Organic matter transport and retention have been largely examined at the reach level in perennial tributaries over short periods (<24 h; but see Chadwick and Huryn 2005, Chadwick et al. 2010), and the primary interest regarding instream retention is because this is where most biological processing is expected to occur (Tank et al. 2010). Wohl et al. (2017) proposed a conceptual framework to examine organic carbon flux through river networks that incorporates climate, network position, and river corridor confinement. They indicated that headwater tributaries that drain steep landscapes have minimal potential for organic carbon storage because of their relatively confined corridors. The seasonal input of leaf litter, rapid downstream transport, and biological processing within perennial streams may cause food limitation for some consumers by late spring (Richardson 1991, Haapala and Muotka 1998). We counter that the combination of structural complexity (Gooderham et al. 2007) and ephemeral flow regime is likely to make headwater tributaries draining even steep, temperate forest catchments important storage zones for river networks. In mesic regions, runoff generation is more likely to occur in the late winter–spring, several months after peak deciduous leaf input, when soil moisture storage is high and evapotranspiration is low (Buttle et al. 2012, Zimmer and McGlynn 2017). Runoff in ephemeral tributaries may then transport leaf litter that was stored in dry channels to downstream waters, supplying leaf litter when it may be a limiting resource to some downstream consumers.

The purpose of our study was to measure and examine relationships that describe the extent and timing of CPOM deposition and transport from ephemeral tributaries to a downstream perennial stream. We predict that ephemeral tributaries function as a lag in drainages of mesophytic forest watersheds, such that the hydrologic regime and other storage properties (e.g., channel roughness, morphology) of ephemeral tributaries prolong terrestrial CPOM export from forested catchments long after autumnal leaf abscission. We then use our data and previously published data in a series of simple calculations to estimate the cumulative annual leaf export from ephemeral tributaries to a downstream water. We find strong evidence that the ephemeral tributaries in a temperate forest are functionally connected to a downstream water through a delayed CPOM subsidy (lag function) maintained by the asynchrony of autumnal leaf input and late-winter and early-spring pulsed flows.

## METHODS

### Study area

**Fig. 1.**
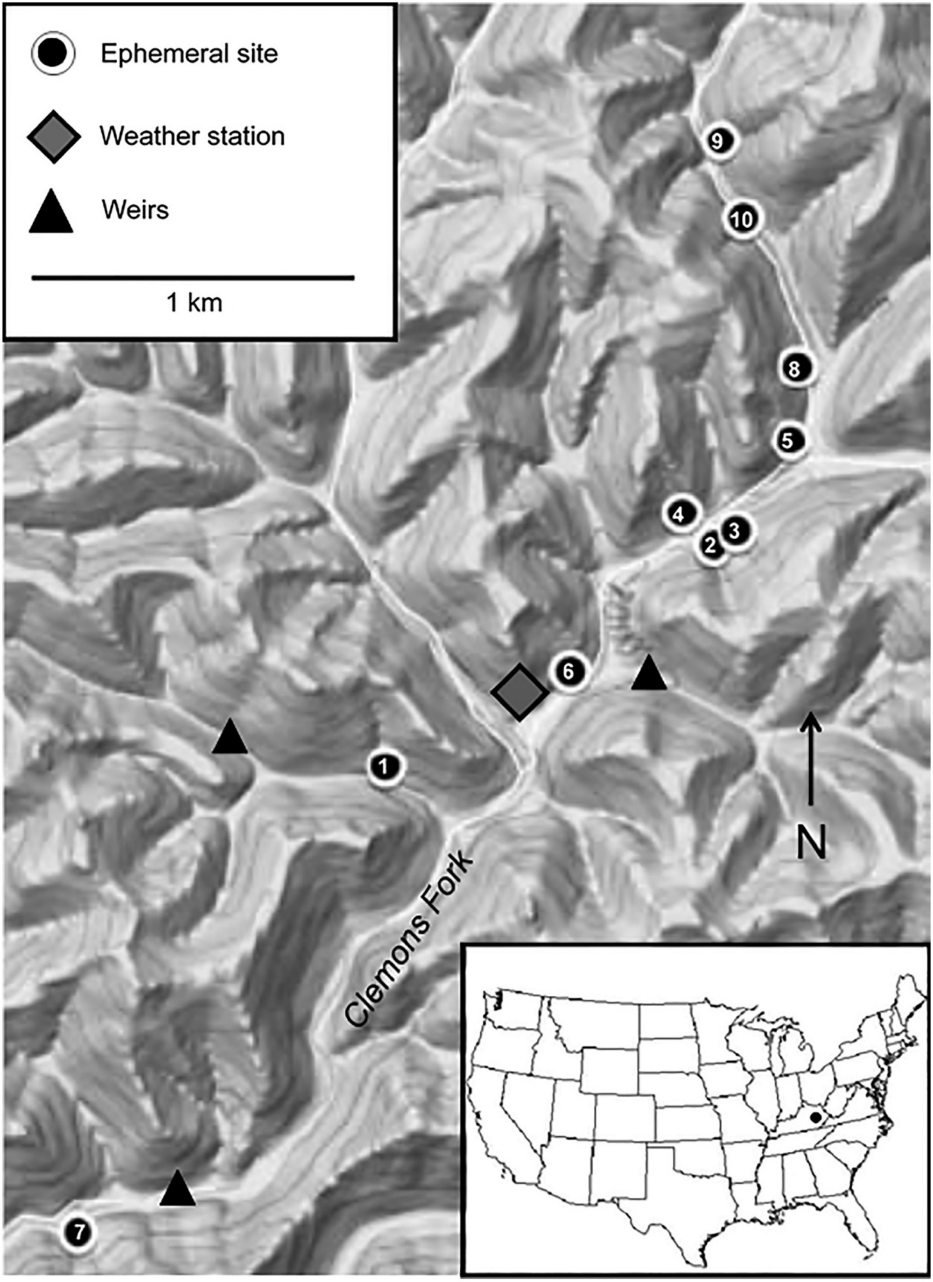
Map of study streams within the Clemons Fork catchment, University of Kentucky’s Robinson ResearchForest in Kentucky, USA.

The study sites were in 10 ephemeral tributaries of the perennial, third-order stream, Clemons Fork (15 km^2^), within the University of Kentucky’s Robinson Forest (37°27^’^N, 83°08^’^W) in eastern Kentucky, USA (Table 1, Fig. 1). The Clemons Fork catchment is within the Cumberland Plateau physiographic section and the Central Appalachian level III ecoregion (Woods et al. 2002) and is characterized by finely dissected topography with narrow ridges and steep hillslopes where 80% of the land area has slopes >30% (Cremeans and Kalisz 1988). Climate is humid and temperate with ~120 cm of precipitation per year. While rainfall is fairly uniformly distributed throughout the year, it tends to be highest in spring and lowest in summer, and snow contributes <5% of annual precipitation (Coltharp and Springer 1980, Coltharp and Albright 1987). Soils were derived from interbedded sandstones, siltstones, shales, and coal geology. The soils graded from well-drained, loamy colluvium and residuum with rock outcrops in the upper hillslopes to well-drained, sandy–loamy colluvium on the lower slopes to sandy–silty alluvium along Clemons Fork (Hayes 1998). The forest was predominantly second-growth upland hardwood and mixed mesophytic (harvested between 1890 and 1920) with oaks (*Quercus* spp.), hickory (*Carya* spp.), yellow poplar (*Liriodendron tulipifera*), and beech (*Fagus grandifolia*) among the dominant overstory species (Phillippi and Boebinger 1986, Reece and Krupa 2013). The physiography, chemistry, and biology of the Clemons Fork catchment are used as a state reference condition and have been shown to be similar to other forested catchments in the region (Carpenter and Rumsey 1976, Coltharp and Albright 1987, Ormsbee and Khan 1989)

The relatively shallow soils, steep slopes, and lateral subsurface and macropore flow in Clemons Fork catchment produce rapid recharge and flashy hydrologic response to precipitation (Coltharp and Springer 1980, Ormsbee and Khan 1989, Williamson et al. 2015). Coltharp and Springer (1980) estimated that stormflow represented 44% of the annual discharge from a Clemons Fork tributary. The dissected landscape of the Clemons Fork catchment has an extensive network of stream channels in which the presence of surface flow greatly expands and contracts (Johnson et al. 2010). Ephemeral tributaries represent 54–57% of the channel length within the Clemons Fork network (Fritz et al. 2013, Villines et al. 2015). Previous research in nearby ephemeral tributaries of Clemons Fork documented surface flow occurring primarily between mid-November and mid-May with only one or two events between mid-May and mid-November (Fritz et al. 2010); however, Williamson et al. (2015) measured water to be present for long periods between May and December in places along two other headwater tributary reaches expected to be ephemeral. Regardless, we expect that ephemeral tributaries are most commonly to have surface flows of sufficient magnitude to transport organic matter to the downstream network in the spring months.

### Physical habitat characterization

A weather station located in a maintained grassland opening within the Clemons Fork catchment recorded precipitation at 15-min intervals with a tipping bucket linked to a Campbell Scientific CR10X data logger (Fig. 1). Precipitation events were identified as successive intervals of rain separated by at least one 15-min period with no rainfall, and the minimum duration of a precipitation event was 15 min. No precipitation data were collected from the Robinson Forest weather station from 14 January to 1 February 2014. Daily precipitation values for the missing dates were derived from a fitting a relationship (*r*^2^ = 0.85, *n*= 321) between daily precipitation data from the Robinson Forest weather station and daily precipitation data from Hindman, Kentucky (http://www.kymesonet.org/historical_data.php), approximately 21 km southeast of the Robinson Forest weather station. Flow on the mainstem of Clemons Fork and two tributaries was measured in weirs (10:1 and 3:1 side-sloped broad-crested combination weirs for Clemons Fork and the tributaries, respectively) equipped with miniTROLL or Level TROLL data-logging pressure transducers (In-Situ, Ft. Collins, Colorado, USA; Fig. 1). The pressure transducers took measurements on 15-min intervals and were downloaded monthly.

A 1–2 m long reach was delineated for each ephemeral tributary immediately upstream from the flood-prone area of the downstream channel in November 2013 (following leaf abscission). Channel width, slope, and the percent of the streambed containing cobble and boulder substrates were measured for each study reach. While all streams contained or were spanned by large wood (>10 cm diameter), no large wood was in the study reaches. The channel origins were identified in the field and used to determine total channel length for each stream (Fritz et al. 2006). Electrical resistance data loggers (Fritz et al. 2006) were deployed in each reach to characterize the duration, frequency, and timing of dry periods. The data loggers were positioned to record status in the thalweg at the upstream end of each study reach. Data from the data loggers were retrieved every month (December 2013–May 2014). The loggers record the time-associated binary flow state (dry or wet) changes. Flow state data of each channel were converted to 15-min intervals to align with the precipitation and discharge data, limiting the minimum duration of flowing or dry periods to 15 min.

### CPOM deposition and surrogate transport

Abscised *Ginkgo biloba* leaves were collected in Cincinnati, Ohio, counted into batches of two hundred leaves, then placed into resealable plastic bags and stored in a refrigerator (1.5°C). Wooden dowels (*L. tulipifera*; 1.3 cm diameter) were cut to uniform lengths (26.7 cm), soaked in water for 48 h, and spray-painted orange. All the naturally occurring CPOM was removed by hand from the active channel within each stream reach at the start of the study. A batch of Ginkgo leaves and 10 wooden dowel rods were distributed evenly within the active channel of each study reach. After ~1 month (26–36 d), any dowel rods remaining within the reaches were counted and all CPOM (including any remaining Ginkgo leaves) within the reaches was placed within a resealable plastic bag and returned to the laboratory. Any dowels found downstream were also collected. New batches of Ginkgo leaves and dowel rods were then redeployed within the study reaches. Surrogate deployment and sample collection were repeated for six consecutive periods in 2003–2004 spanning autumn to spring (November–December, December–January, January–February, February–March, March–April, and April–May).

In the laboratory, any retained Ginkgo leaves were removed from the collected organic matter and counted. Based on the number of retained Ginkgo leaves and dowel rods, we calculated the percent exported from the reaches per day for each month. The natural CPOM was sorted into leaves (broad and needle) and wood (twigs, bark, nuts, and cones), dried (70°C) for >48 h, weighted, combusted at 550°C for ≥2 h, and reweighed to determine ash-free dry mass (AFDM). When large amounts of leaves or wood were collected, subsamples were taken to determine the percent AFDM of the entire sample. Using the natural CPOM collected from each reach, we calculated the daily CPOM deposition rates for each month. Because the study took place after leaf abscission, we attribute most of the deposition of native leaves and wood within the study reaches to fluvial transport from upstream reaches of the ephemeral tributaries. However, we cannot completely discount that some of the deposited organic matter originated from litterfall or wind-driven lateral transport directly into the stream channel.

### Data analysis

Variation in ephemeral tributary export (Ginkgo leaves and dowel rods) and deposition (leaves and wood) across time periods was compared using one-way ANOVA (PROC GLM, SAS 9.4; SAS Institute, Cary, North Carolina, USA). Multiple comparison tests (LSMEANS, Tukey’s adjustment) were used to determine where specific differences resided if significant differences were found among the six time periods. Pearson’s correlations were used to assess relationships between flow duration at ephemeral tributaries and CPOM export and deposition. Normality was confirmed using the Shapiro–Wilk test, whereas residuals were plotted to assess inequality of variance. Data were log-transformed when they did not meet statistical assumptions.

We used a combination of existing data and data from this study to empirically model the cumulative export of leaf litter from ephemeral tributaries to Clemons Fork. We used the best estimate of ephemeral channel length from Fritz et al. (2013) and the average ephemeral channel width from this study to calculate total contributing ephemeral channel area (assumed even tapering of the channel area by dividing calculated area by half). Annual litterfall was estimated from published values measured within the Clemons Fork watershed (Newman et al. 2006, Littlefield et al. 2013). Annual lateral leaf input to the channels was estimated to be 25% of the litterfall (Benfield 1997). We estimated standing crop within ephemeral channels to be the sum of litterfall input and lateral input minus 15% for losses from leaching and breakdown within the ephemeral channels (Richardson 2000). We used our monthly estimates of proportional leaf export rate to iteratively estimate the cumulative export of leaf litter from ephemeral tributaries to Clemons Fork. Our simple model assumes negligible export from ephemeral tributaries between mid-May and mid-November (when the channels are most likely to be dry), uniform leaf retention along the channels, and leaf input after mid-November was negligible.

## RESULTS

### Meteorological and hydrological patterns

**Fig. 2.**
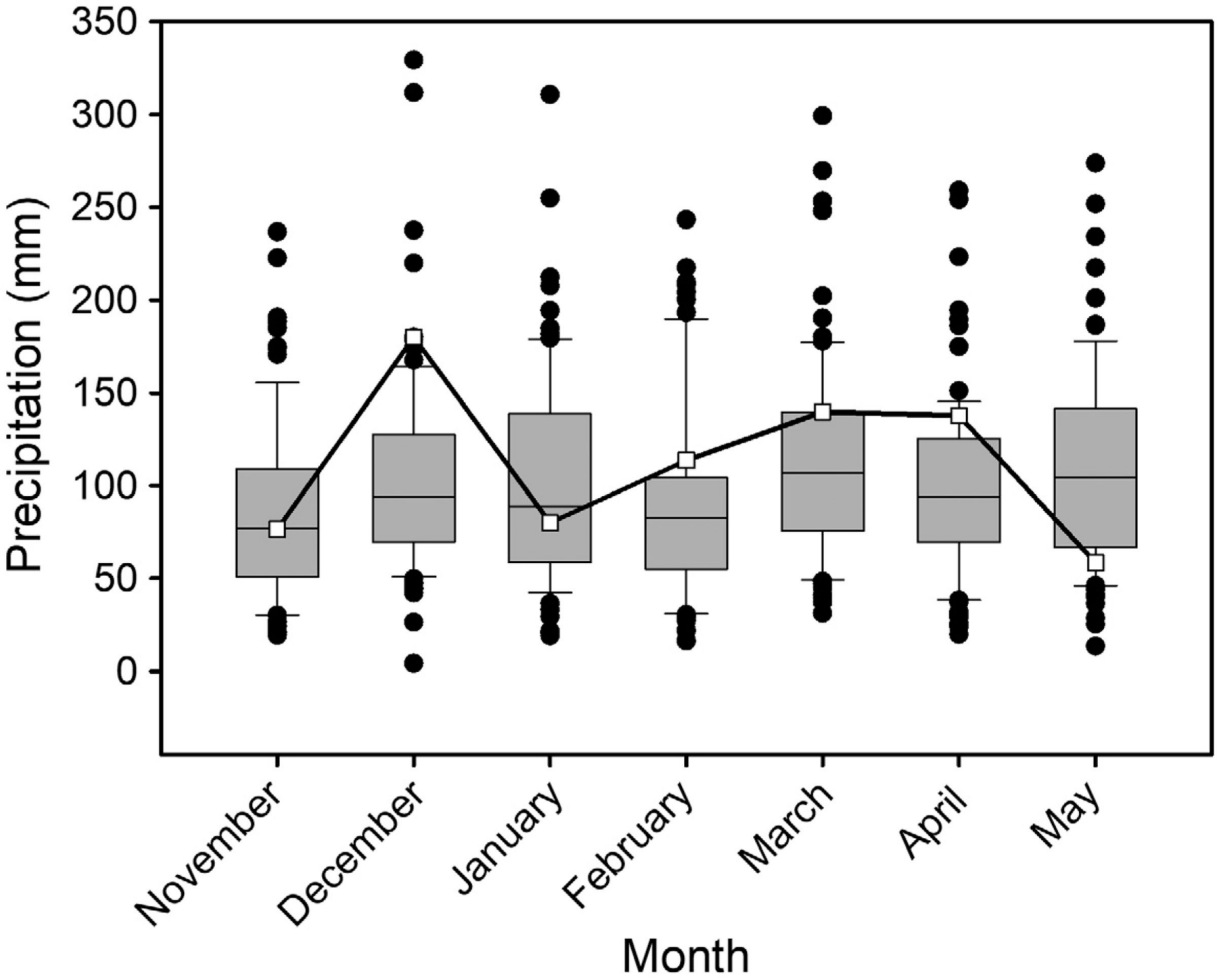
Monthly rainfall totals during the study period (open squares; November 2013–June 2014) and across the period of record (box plots; 1899–2014), Jackson, Kentucky, USA. Line inside boxes are medians, box ends are quartiles, whiskers show the 90th and 10th percentiles, black circles show years outside the 90th and 10th percentiles.

**Fig. 3.**
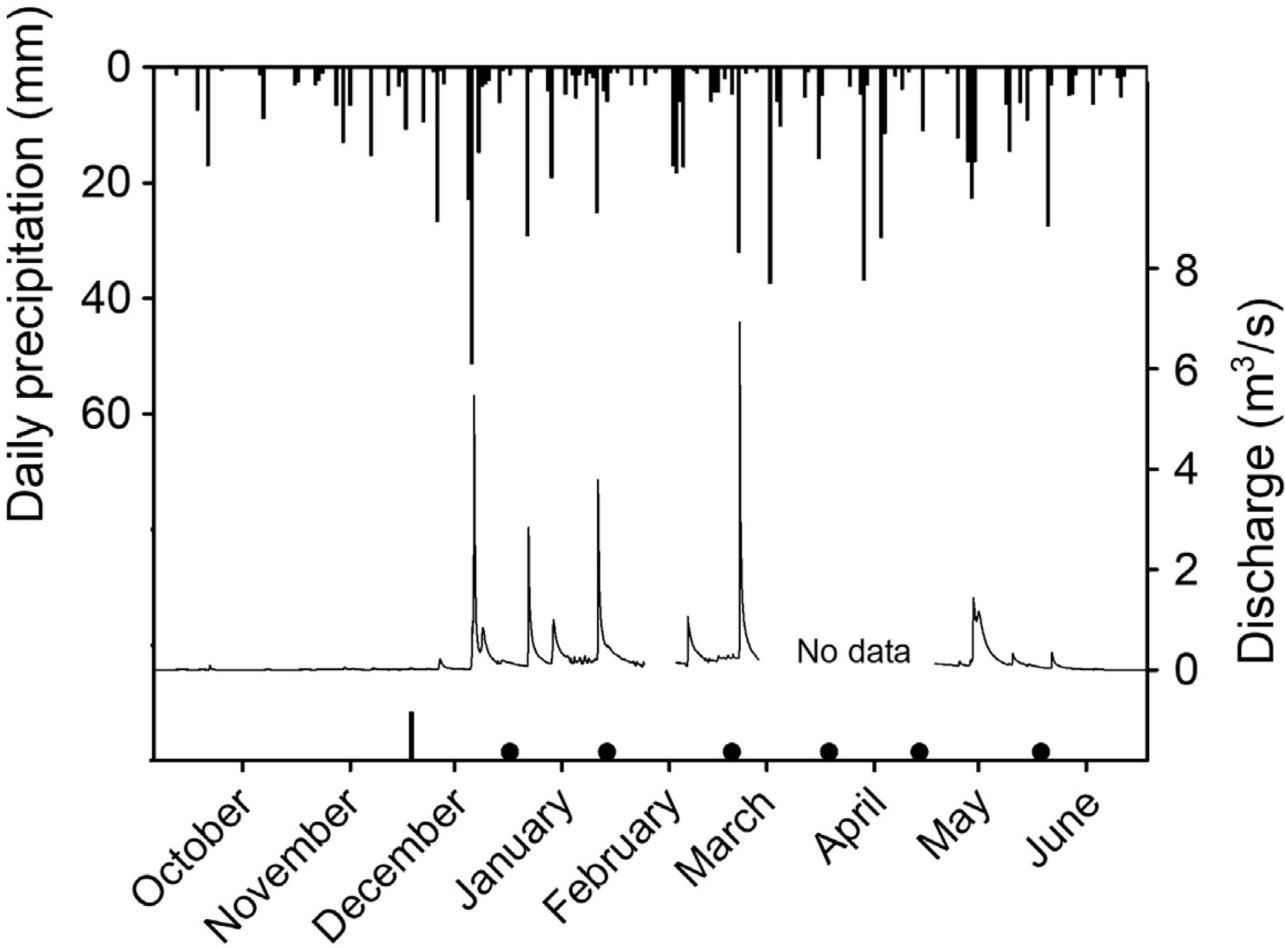
Daily precipitation and discharge at the mainstem weir on Clemons Fork, Kentucky. Black circles on *x*-axis show organic matter collection dates, and the black bar identifies initial deployment.

Precipitation during the study was normal to slightly above normal compared to historical data (Fig. 2). Precipitation frequency recorded at Robinson Forest was regular, with 55% of rain days being associated with 2–5 consecutive days with rain. Flow in Clemons Fork remained low through the fall season, and storm flows did not occur until December (Fig. 3). The longest consecutive period without rain was 7 d, and this occurred once in March and once in May (Fig. 3).

The number of precipitation events ranged from 33 to 83 per study period (Table 2). The heaviest rain events and the highest number of heavy rain events (>10 mm) were in December–January, February–March, and March–April, whereas the magnitude of rain events was mildest in May–June (Table 2, Fig. 3).

**Fig. 4.**
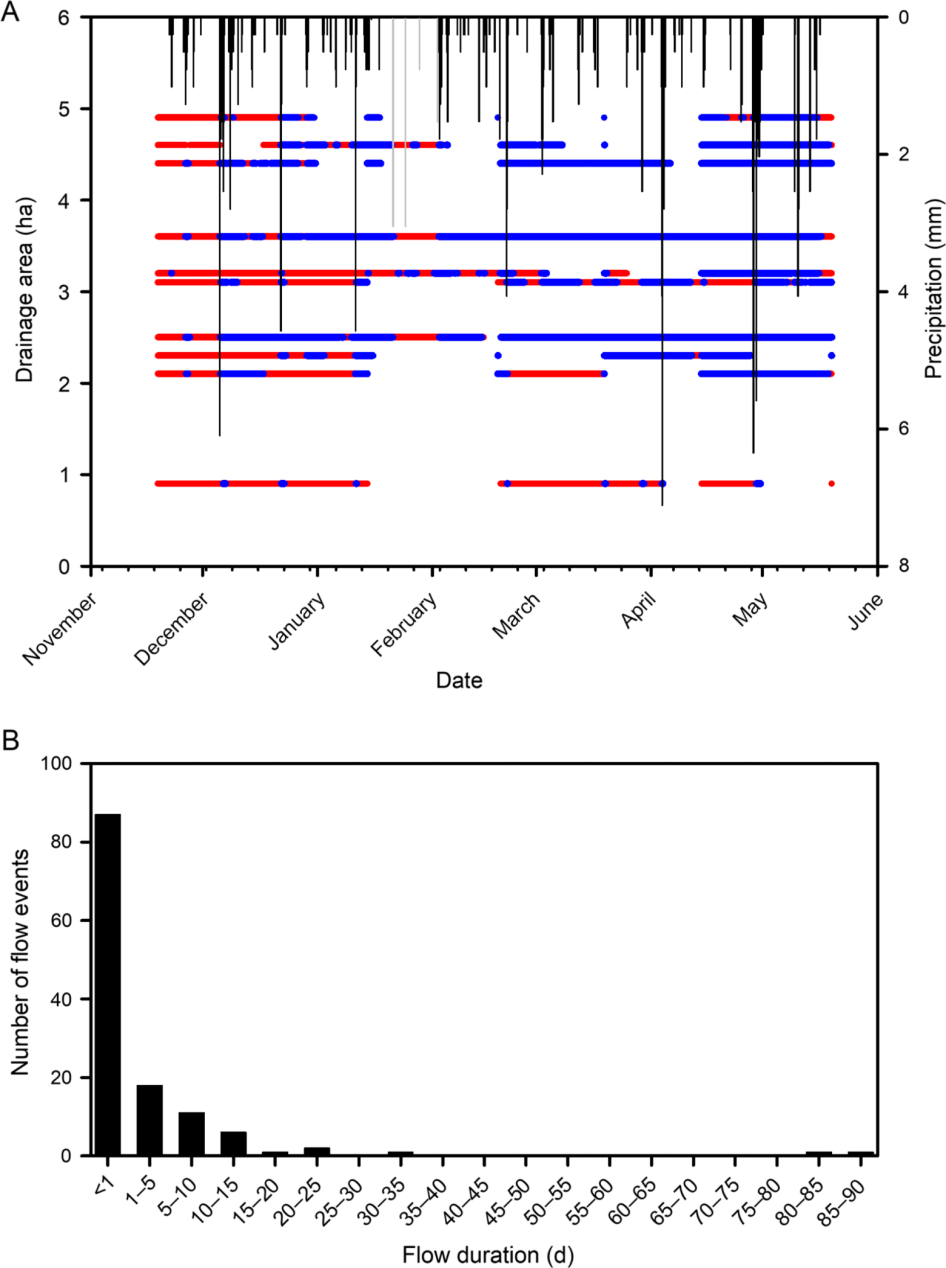
Flow conditions (red is dry and blue is wet) at the 10 ephemeral tributaries by their drainage area during the study (A). Gaps are periods when data loggers malfunctioned. Precipitation is shown at 15-min intervals (in black) except 14 January–1 February where daily precipitation is shown (in gray). Distribution of 128 complete flow events recorded across 10 ephemeral tributaries to Clemons Fork, Kentucky, during November 2013–June 2014 (B). Flow data were missing from 38.3% of the dataset, including 15–28 partially recorded flow events not shown.

Precipitation events often coincided with the onset of flow recorded by data loggers in the ephemeral tributaries (Fig. 4A). The number of recorded flow events and the percent of record with flow (Table 3) had positive relationships with drainage area (Pearson’s *r*= 0.69, *P*= 0.027 and *r*= 0.52, *P*= 0.126, respectively) such that tributaries with larger drainage areas tended to have more flow events and tended to flow more often than those with smaller drainage areas (Fig. 4A). The maximum recorded flow duration was unrelated to tributary drainage area (*r*= −0.03, *P*= 0.943). Flow frequency and duration in tributaries draining catchments with similar aspects varied as widely as those with different aspects. Data logger malfunction resulted in 39% of the flow data missing across all ephemeral tributaries during the study and ranged from 0% to 55.5% among individual tributaries (Table 3). A total of 128 complete flow events (recorded the onset of flow and drying) were recorded across the 10 tributaries during the study, and 68% of those events had durations lasting less than one day (Fig. 4B). However, continuous surface flow recorded at two of the tributaries lasted for ≥80 d (Fig. 4B, Table 3).

### CPOM deposition and surrogate transport

**Fig. 5.**
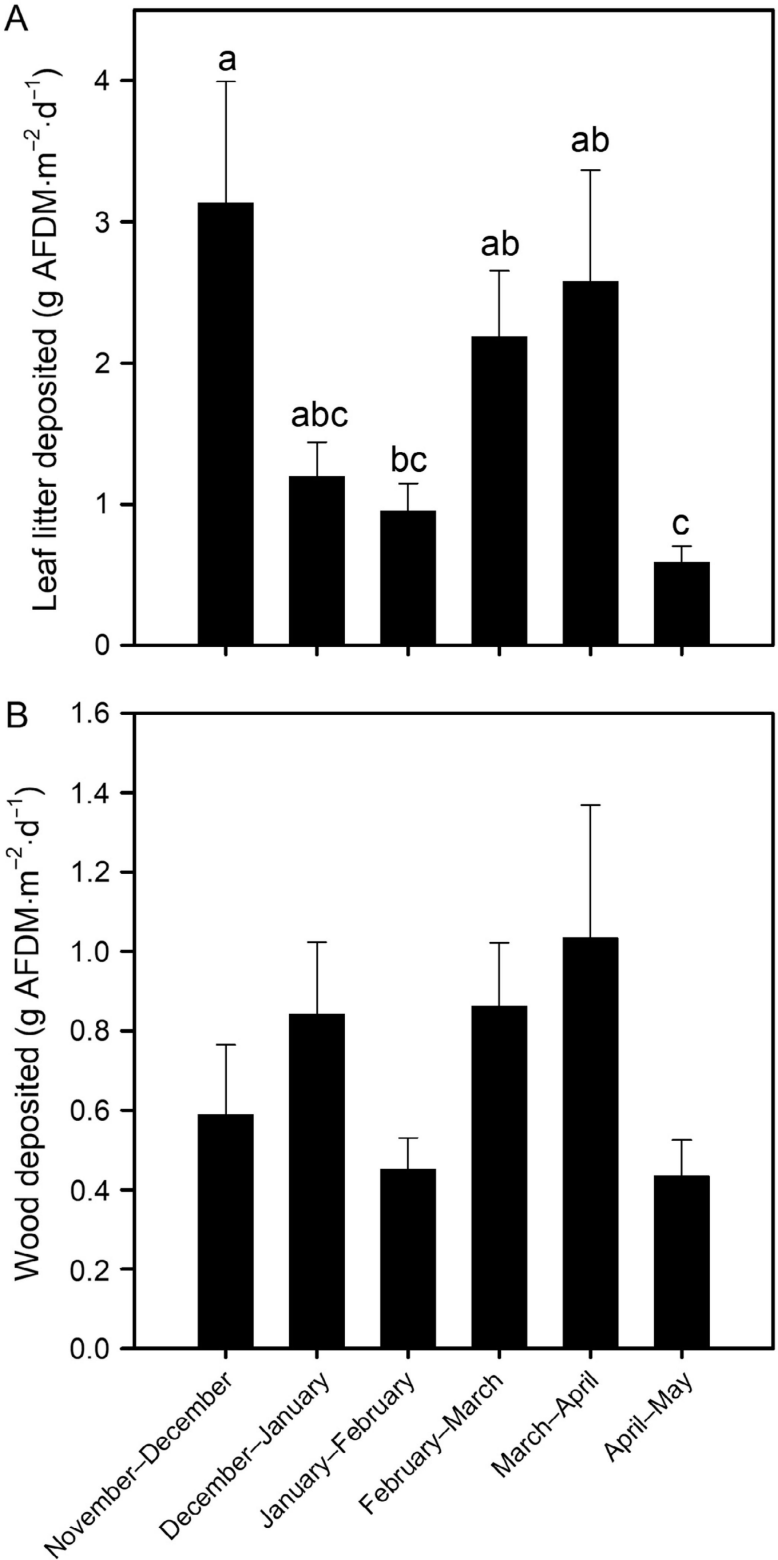
Mean (±1 SE) mass of leaves (A) and wood (B) deposited in study reaches during each 1-month period. Bars with the same letters are not significantly different (adjusted Tukey’s post hoc test, *P*>0.05).

Natural leaves represented most of the deposited CPOM measured in ephemeral reaches in 81.7% (49 of 60) of the measurements. The amount of transported and deposited leaves into reaches varied across periods (Fig. 5A; *F*_5,54_ = 5.84, *P*= 0.0002, *r*^2^ = 0.35), but the amount of wood did not differ (Fig. 5B; *F*_5,54_ = 1.99, *P*= 0.09, *r*^2^ = 0.16). The amounts of natural leaves and wood deposited in ephemeral reaches in February–March and March–April were comparable to those in November–December. The amount of deposited leaves did not differ among streams (*F*_9,50_ = 1.70, *P*= 0.11, *r*^2^ = 0.23), but differences in deposited wood was detected among streams (*F*_9,50_ = 3.89, *P*= 0.0009, *r*^2^ = 0.41). Tributaries E1 and E7 had lower amounts of deposited wood than in E5 and E7 had lower amounts than in E9.

**Fig. 6.**
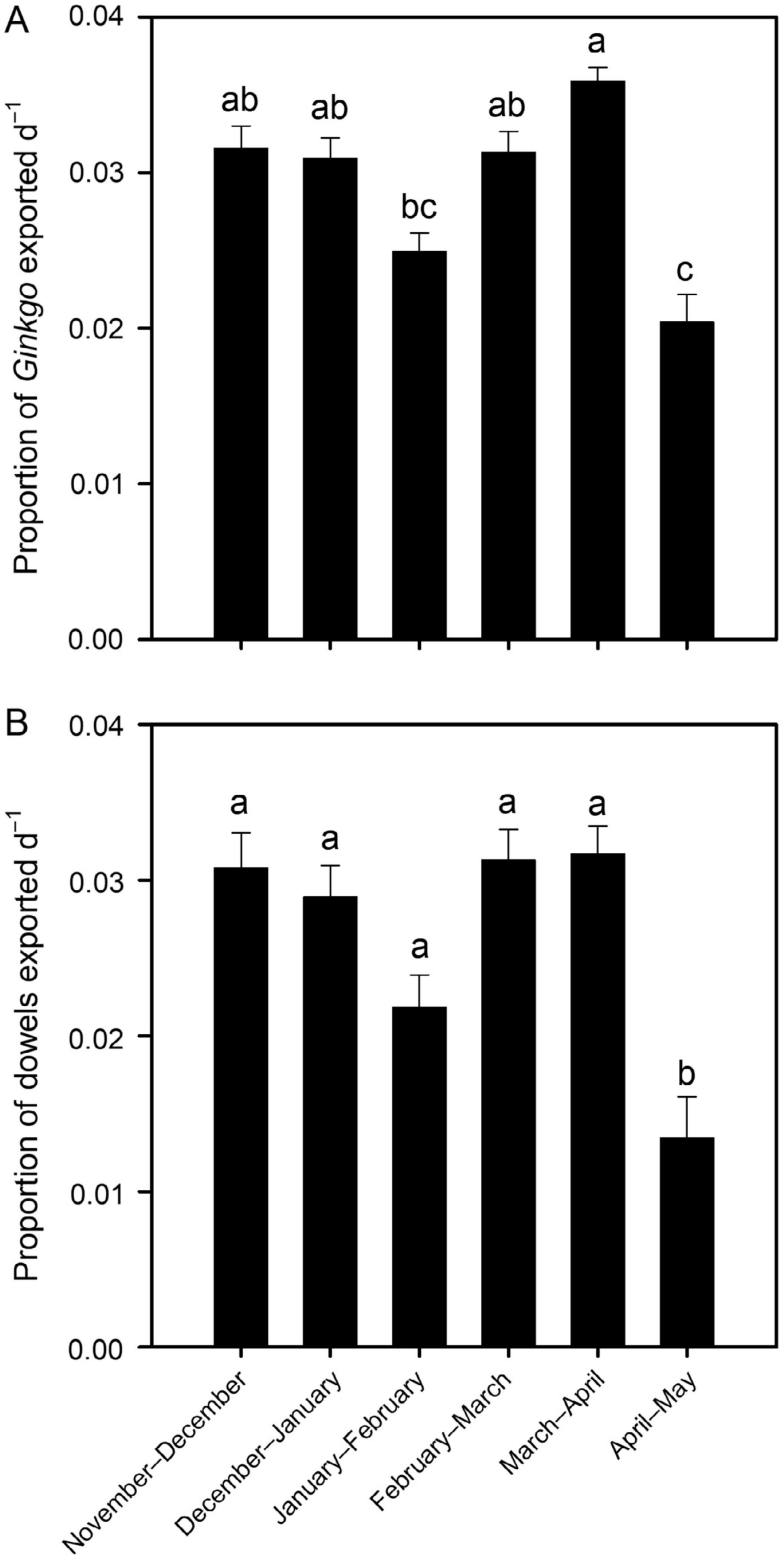
Mean (±1 SE) proportion of deployed Ginkgo leaves (A) and dowel rods (B) exported downstream per day during each 1-month period. Bars with the same letters are not significantly different (adjusted Tukey’s post hoc test, *P*>0.05).

The percentage of deployed Ginkgo leaves and dowel rods exported downstream ranged from 28% to 100% and 0% to 100%, respectively. All of the deployed leaves (200 per reach) and dowels (10 per reach) were exported downstream in respectively 8.3% and 33.3% of the periods across the 10 ephemeral reaches studied. The proportion of Ginkgo leaves and dowel rods transported from the reaches per day varied across deployment periods (Fig. 6; *F*_5,54_ = 12.91, *P*<0.0001, *r*^2^ = 0.54 and *F*_5,54_ = 10.66, *P*<0.0001, *r*^2^ = 0.50, respectively). The Ginkgo leaf transport rate was highest in March–April period and lowest in the April–May period (Fig. 6A). The dowel transport rate in April–May was lower than all other study periods (Fig. 6B). Transport rates for Ginkgo leaves and dowels were positively correlated (*r*= 0.82, *P*<0.0001, *n*= 60). Wood and leaf transport rates were not correlated with their respective deposition amounts. Ginkgo leaf transport, dowel transport, and wood deposition rates were also not correlated with recorded flow duration or frequency. Leaf deposition rates had a weak negative correlation to recorded flow duration (*r*= −0.37, *P*= 0.026, *n*= 37) and were not correlated with flow frequency. Mean amounts of deposited leaves within periods were correlated to the maximum daily rainfall within periods (*r*= 0.94, *P*= 0.005, *n*= 6).

**Fig. 7.**
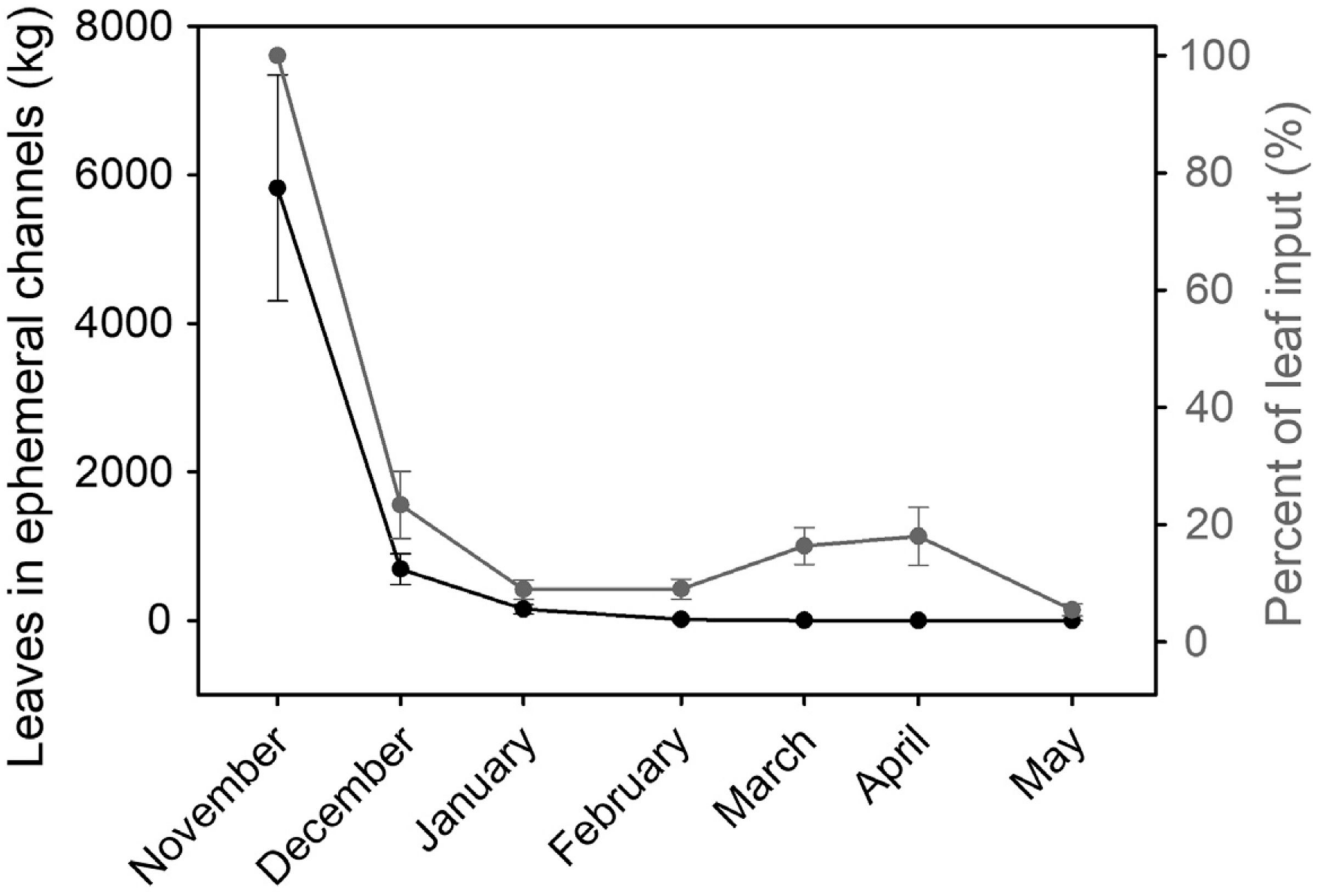
Predicted mean mass of leaves (black line) within 52.64 km of ephemeral channel within the Clemons Fork network (Kentucky, USA) and the mean mass of leaves deposited (gray line) measured from 10 ephemeral tributaries over time relative to the leaf input estimates (Newman et al. 2006, Littlefield et al. 2013). Error bars are 95% confidence intervals.

The total length of ephemeral tributaries within the Clemons Fork network was estimated to be 52.6 km or 56.9% of the total length (Fritz et al. 2013). Using the average channel width of 0.57 m, we estimate the total ephemeral channel area to be 15,002 m^2^ or 0.14% of the Clemons Fork watershed area. We estimate the mean total annual leaf input into ephemeral channels to be 388.0 g AFDM·m^−2^·yr^−1^ or 5821.3 kg/yr across the entire ephemeral channel area within the Clemons Fork network (Appendix S1: Table S1). Using the monthly proportional leaf export rates, we modeled the cumulative downstream export of leaves from ephemeral channels (Fig. 7). The model predicted exponential loss of leaf standing crop with nearly all the leaves in ephemeral tributaries to be exported by mid-May, resulting in a cumulative mean annual leaf export of 110.6 kg AFDM·[km ephemeral tributary]^−1^·yr^-1^. However, the modeled pattern of leaf export does not align with the mean pattern of deposited natural leaves within the ephemeral tributaries which had a bimodal pattern (Fig. 7) which indicates a more delayed leaf subsidy.

## DISCUSSION

The upstream extent of surface flow in most river networks varies over time in response to changing seasons and individual precipitation events (Roberts and Archibold 1978, Wigington et al. 2005). Much of the expansion and contraction of surface flow in networks occurs in ephemeral tributaries. The majority of our understanding on the hydrology of ephemeral channels is based on studies in arid regions (Lane et al. 1971, Camarasa-Belmonte and Segura-Beltrán 2001, Morin et al. 2009), despite widespread occurrence in mesic regions (Datry et al. 2014). As shown in our study, the hydrology of ephemeral headwater streams in temperate forests is characterized by mostly short duration flows initiated by precipitation events. However, these flow events are very frequent during winter and spring (Table 3). The number of flow events in ephemeral tributaries in the Ouachita Mountains, AR, ranged from 2 to 39 per year over a 5-yr period (Miller et al. 1988). The present study documented substantial variation in the duration of recorded surface flow across the study tributaries, ranging from <1 to >80 d of continuous surface flow. In another study at Robinson Forest, six other tributaries with similar drainage areas (1.0–3.3 ha) as those in the present study had flow between 53% and 67% of the time over a 13-month period (Svec et al. 2005).

While there were periods when all the study tributaries were dry or were flowing, more often during the study there were some tributaries with surface flow and some without surface flow (Fig. 4A). Variation in precipitation among sites is likely to be minor because of their similar elevations and close proximity. Moreover, convective storm events that do account for precipitation deviation across the forest do not generally occur during the winter and spring months when the study was conducted. Unaccounted for variation in water storage and flow paths that link hillslopes to ephemeral tributaries (Sidle et al. 2000) likely contributed to the differences in the recorded runoff response among ephemeral tributaries. Because we recorded surface flow frequency, duration, and timing near the base of the ephemeral tributaries, it is possible that we did not capture the hydrologic character of the entire ephemeral tributary as surface flow can be discontinuous in these channels. Several studies that have mapped flow in tributaries have documented various degrees of interrupted or fragmented surface flow where upstream reaches with surface flow are interrupted by downstream reaches lacking surface flow (Hunter et al. 2005, Godsey and Kirchner 2014, Shaw 2016). This spatial variation in how hydrologic connections are expressed as surface flow in headwater tributaries has strong implications on their management and the implementation of policy. Flow characteristics of tributaries are “evaluated at the farthest downstream limit” of tributaries according to the guidance for jurisdictional determinations under the Clean Water Act in the United States (USACE and USEPA 2008). Debris flow deposits, bedrock fractures, woody debris, and vegetation can locally affect channel form such that hydrologic connections may not be expressed as longitudinally continuous surface flow (Zimmerman et al. 1967, Jensen et al. 2017). Because of the discontinuous nature of surface flow in many tributaries, the guidance continues by stating “the flow regime that best characterizes the entire tributary should be used” to determine whether a tributary is a relatively permanent water (USACE and USEPA 2008).

The pattern of CPOM deposition and surrogate transport in this study indicates that ephemeral tributaries store and continue to export leaf litter and small wood to downstream waters several months after peak leaf abscission. This asynchronicity in the timing of leaf abscission (CPOM input) and surface flow drives the lag function we observed in the headwater ephemeral tributaries. Rather than duration and frequency of flow, the cumulative amount of leaf litter transported and deposited within the study reaches each month was related to peak magnitude of rainfall. Although we did not directly measure runoff magnitude, it is a reasonable premise that runoff magnitude in ephemeral tributaries during the dormant season should reflect measured rainfall magnitude. Although often difficult to directly measure, large storms are key periods of CPOM export in streams. In three perennial tributaries in North Carolina, 63–77% of CPOM was exported during storm flows that represented <1% of the study period (Wallace et al. 1995). The first flush or flood bores associated with the often, sudden onset of flow in intermittent rivers can transport large quantities of organic matter and other materials that had accumulated while the channels were dry (Jacobson et al. 2000, Corti and Datry 2012).

More research has focused on the storage and preconditioning of CPOM by lateral features, such as gravel bars, banks, and floodplains, that are seasonally or ephemerally inundated (Gurtz et al. 1988, Merritt and Lawson 1992, Hutchens and Wallace 2002, M’Erimba et al. 2007, Langhans et al. 2013, Riedl et al. 2013) than by ephemeral tributaries. The substantial amounts of leaf litter estimated to be transported from ephemeral channels in this study suggest that organic matter processing within ephemeral channels is limited. The breakdown of white oak leaves (October–April) was significantly slower in ephemeral reaches (mean *k*= −0.0025) than in intermittent (−0.0071) and perennial (−0.0054) reaches in Robinson Forest streams (Fritz et al. 2010). Riedl et al. (2013) attributed the slower decay of leaf litter on dry stream banks compared to leaf litter on wet streambed to the availability of the litter to stream consumers. Leaf litter breakdown was also slower on banks than on streambeds in second-order perennial streams in North Carolina, but leaf packs on banks in that study were colonized by stream consumers but fewer in number and biomass than leaf packs in the streams (Hutchens and Wallace 2002). In contrast, the breakdown of deciduous leaves on floodplain soils in Michigan during the spring was rapid, where 95% of total annual leaf fall was found to be consumed by earthworms within a four-week period (Knollenberg et al. 1985). The combination of ephemeral and delayed hydrologic connection relative to leaf abscission, the highly dissected topographic setting, low moisture conditions, and low flow competence is likely to make ephemeral tributaries at least as important as lateral features in functioning as lag depositories of CPOM to Clemons Fork in this study and similar perennial streams.

The importance of ephemeral tributaries as lag depositories of CPOM in the network is likely to be substantial in temperate regions with deciduous vegetation compared to other regions where the timing of flow and leaf input may not be asynchronously timed (e.g., tropical forests) or where allochthonous CPOM subsidy is minor (e.g., tundra). Changes in climate and land use that result in aligning the timing of leaf abscission with surface flow in headwater tributaries will reduce the lag function of headwater tributaries. Increasing the magnitude and occurrence of runoff in the autumn and early winter are likely to reduce CPOM subsidy from ephemeral tributaries later in the spring when CPOM may be limiting to downstream consumers. For example, warming climate has resulted in earlier flooding throughout Europe over the last 50 yr (Bloschl et al. 2017). Urbanization results in more€ frequent and intense wet weather flows than natural land cover (Shuster et al. 2005) and potentially year-round flows through effluent discharges and/or elevated groundwater tables that would increase in-channel breakdown rates (Crippen and Waananen 1969, Passarello et al. 2012). Retention of long-term leaf analogs was negatively related to the percent of impervious surface area among tributary streams in Florida (Chadwick et al. 2010). Land use and climate changes that increase the coincidence of surface flows with leaf abscission remove the lag function of ephemeral tributaries that could provide to downstream waters.

Because of their small size, individual headwater ephemeral tributaries are unlikely to have a large impact on a mainstem (Rice 2017), but because of the vast numbers of tributaries that drain into a mainstem, the cumulative contribution can be significant (Freeman et al. 2007). We used data from this study and previous studies to estimate the cumulative CPOM contribution from ephemeral tributaries and arrived at a cumulative annual leaf export of 110.59 kg AFDM·km^−1^·yr^-1^. Among the more common leaf consumers in the Clemons Fork network is the limnephilid caddisfly, *Pycnopsychegentilis* (42.3% of stream insect biomass during the spring; K. M. Fritz, *unpublished data*). Previous studies have found larvae of this species consume leaf litter almost exclusively throughout the year despite less leaves available for later instars in the late spring (Hutchens et al. 1997). Based on previously published relationships between standing crop of leaf litter and abundance and biomass of *P. gentilis* (Eggert and Wallace 2003), we estimate the cumulative contributions of leaves from ephemeral tributaries to Clemons Fork to support 329.3 individuals/m^2^ and 188.7 mg AFDM/m^2^ of *P. gentilis*. Previous studies suggested that slower decaying species of leaves may be important to supplying consumers with leaves in spring and summer when faster decaying leaves are gone (Cummins et al. 1989). We submit that in addition to the retention of slower decaying leaves, that the lag function of ephemeral tributaries provides a delayed subsidy of CPOM to downstream consumers.

Most organic carbon is exported from headwater streams as dissolved organic carbon (DOC; Tank et al. 2010) and DOC concentration often increase during storm flows (Meyer and Tate 1983, Buffam et al. 2001, Inamdar et al. 2011). Leachate from leaf litter stored in stream channels can be a significant source of DOC to downstream waters (Meyer et al. 1998). Based on the relationship between leaf litter standing crop and DOC concentration described by Meyer et al. (1998) in two Appalachian headwater streams and our estimated leaf litter standing crop in ephemeral channels, we estimate that up to 1.19 mg DOC/L from leaf leachate is exported from ephemeral tributaries during runoff events. This is likely a high estimate as more leaves within perennial flowing tributaries are likely to be completely inundated longer than leaves within ephemeral tributaries. Regardless, in addition to being functionally connected to downstream waters as a CPOM lag, ephemeral tributaries are likely also functionally connected as transformers of CPOM that is exported as DOC to downstream waters.

There were discrepancies between our predicted pattern of CPOM standing crop and our measured pattern of deposited leaves in the ephemeral tributaries (Fig. 7). Some plausible explanations for these differences include our use of surrogates to estimate monthly export that do not accurately reflect the dynamics of natural leaves, despite Ginkgo leaves having been successfully used to characterize short-term transport of leaves in perennial streams in other studies (Speaker et al. 1984, Lamberti et al. 2017). The export and deposition dynamics likely vary along ephemeral tributaries (e.g., local differences in channel slope and bed roughness) and so may not have been reflected by those we measured near the tributary mouths. Our simple model also assumes uniform dispersion or the proportion of leaves exported from the ephemeral tributaries over time is equal regardless of their longitudinal distance needed to travel. It is also possible that lateral input may be higher than 25% of litterfall in the steep topography of Robinson Forest (Orndorff and Lang 1981). Our calculations also assumed that lateral input of leaves is restricted to autumn and does not occur during the late winter and spring seasons. Marcescent leaves from beech and oak species (Angst et al. 2017) could also contribute to spring litterfall and lateral input into ephemeral tributaries that our model did not consider. The pattern of leaf deposition clearly indicates a lag in downstream leaf export from the ephemeral tributaries that our simple model underestimates. High upstream retention within the ephemeral tributaries could also result in relatively low leaf deposition within our study reaches, further supporting that ephemeral tributaries are functionally connected as lags to the downstream network. Incorporating the timing of freeze–thaw, runoff, and wind in our calculations may have provided a more realistic pulsed pattern of leaf export from the ephemeral tributaries. Further research on terrestrial–aquatic connections will improve our understanding of the extent to which ephemeral tributaries influence downstream waters.

Annual lateral input of leaves in nine first- to second-order, forested streams in the eastern United States ranged from 89 to 164 g AFDM·m^−1^·yr^−1^, with a mean of 150 g AFDM·m^−1^·yr^−1^(Webster et al. 2006). Our annual flux estimate of 110.6 g·m^−1^·yr^−1^ of ephemeral streams to Clemons Fork is comparable. However, we submit that ephemeral tributaries delay more of their annual CPOM flux during spring than lateral CPOM flux from forested slopes. Lateral leaf input has been shown to be higher in autumn than in spring for some deciduous forests (Fisher and Likens 1973, Miura et al. 2002). In a Massachusetts forest, lateral leaf input to a stream peaked 1–3 weeks after litterfall peak such that 17% of deciduous litter input to the stream was delayed through lateral input but only 4% was delayed by more than a week after peak litterfall (McDowell and Fisher 1976). In contrast, other studies have found substantial deciduous leaf redistribution during winter–early spring, but still show little movement in late spring through summer (Comiskey et al. 1977, Boerner and Kooser 1989). Some of this variation in the timing of lateral leaf input among studies is likely due to differences in climatic conditions (i.e., wind, snow cover, rainfall). Leaf redistribution, on even steep forest slopes, is lowered by moist conditions through enhanced downslope resistance from leaf-to-leaf adherence (Steart et al. 2006, Funada et al. 2009) and soil fungal attachment (Lodge and Asbury 1988). Although we did not directly compare CPOM flux from ephemeral tributaries to flux from lateral slopes, we expect ephemeral tributaries to have a strong role in the CPOM lag connection to Clemons Fork at the watershed scale. We estimate that 56% of our study watershed drains into ephemeral tributaries (with ~47% of ephemeral drainage directly connected to downstream perennial streams and 53% connected first to intervening intermittent reaches), so that most of the lag associated with CPOM storage and transport from forest slopes must still enter perennial streams through ephemeral tributaries.

Ephemeral tributaries have largely been ignored by stream ecologists and terrestrial ecologists despite being common in most regions. Little has been done to assess or even modify methods and criteria specifically for ephemeral tributaries. We propose that surrogate transport and CPOM deposition over time can be useful way to characterize a functional role of ephemeral tributaries and other habitats that are periodically connected to downstream waters. Hill and Brooks (1996) quantified the lag connection of CPOM between headwater wetlands and a downstream water. Summer thunderstorms triggered overland flow to transport the stored CPOM from headwater wetlands to a second-order stream. An ephemeral tributary in the United Kingdom had key moments of influence on a downstream river through the temporal mismatch between sediment transport from an ephemeral tributary and flooding in a mainstem river (Marteau et al. 2017). Despite draining only 1.2% of the river catchment and flowing only 25% of the year, the recently reconnected ephemeral tributary increased annual sediment yield by 65% (Marteau et al. 2017). Experimental releases of sediment in southwestern Washington demonstrated that ephemeral tributaries rapidly transfer fine sediments (<0.063 mm) but provide a lag function for coarser sediments (Duncan et al. 1987). Our findings support these studies which demonstrate that ephemeral tributaries are functionally connected to larger, perennial waters despite not have a year-round hydrologic connection. In fact, the lack of year-round surface water connection contributes to the potential for ephemeral tributaries to provide a delayed subsidy to downstream consumers when CPOM is likely to be limited. We submit that the ecological influence of tributaries and wetlands on downstream water bodies should take into account more than duration and frequency of surface water connections but other dimensions like magnitude and timing.

## Supplementary Material

Sup1

## Figures and Tables

**Table 1.** Characteristics of the 10 ephemeral tributaries to Clemons Fork, Robinson Forest, Kentucky, USA.

Tributary	Catchment area (ha)†	Valley aspect†	Valley slope (%)†	Elevation (masl)†	Channel length (m)†	Channel slope (%)	Channel width (m)	Cobble and boulder (%)

E1	4.9	S	41.4	296	169.5	23.5	0.44	40
E2	4.4	SW	41.6	311	209.2	9.5	0.77	10
E3	3.2	NW	36.9	314	131.0	30.5	1.00	35
E4	2.1	SE	44.6	311	142.0	33.0	0.50	15
E5	2.3	SE	43.3	315	91.8	16.5	0.52	15
E6	3.1	SE	41.8	292	142.8	19.5	0.56	65
E7	2.5	S	49.7	264	67.0	25.0	0.50	10
E8	0.9	E	44.7	328	29.8	23.5	0.50	15
E9	4.6	SW	40.1	339	174.8	9.5	0.51	30
E10	3.5	E	47.0	327	101.2	31.0	0.72	35

† Derived using 10-m digital elevation model.

**Table 2.** Summary of precipitation patterns across the study periods at Robinson Forest, Kentucky, USA.

Description	November-December	December-January	January-February	February-March	March April	April-May	May-June†

Deployment period (d)	28	28	36	28	26	35	29
Total precipitation (mm)	144.3	102.4	91.4	118.1	95.5	116.1	57.15
No. of precipitation events	83	55	37‡	33	34	56	21
No. of precipitation events >10 mm	2	3	2	3	3	1	1
Max. daily precipitation (mm)	51.3	29.2	18.3	37.3	36.8	22.6	27.4

† No coarse particulate organic matter collection.

‡ At least five events missing (all <10 mm).

**Table 3.** Summary of surface flow events captured by electrical resistance data loggers at 10 ephemeral tributaries of Clemons Fork in Robinson Forest, Kentucky, USA.

Tributary	Number of complete flow events captured	Maximum duration of complete flow event captured (d)	Percent of record with flow	Missing data (%)

E1	15	16.3	43.5	55.5
E2	15	9.6	80.5	30.0
E3	18	5.9	30.0	19.7
E4	5	33.0	42.0	33.8
E5	2	4.6	48.8	46.4
E6	20	13.1	38.0	19.4
E7	9	89.2	81.8	2.6
E8	7	0.7	2.8	36.1
E9	26	10.6	59.4	35.7
E10	11	80.7	78.1	0.0
