# Supplementary material for: Coarse particulate organic matter dynamics in ephemeral tributaries of a Central Appalachian stream network
Source: Ecosphere. Author manuscript; Available in PMC 2020 Aug 13. (PMC7425740; doi:10.1002/ecs2.2654)
Supplement: Sup1 [file NIHMS1524621-supplement-Sup1.pdf]

Ecosphere

Coarse particulate organic matter dynamics in ephemeral tributaries of a Central Appalachian stream network

Ken M. Fritz, Gregory J. Pond, Brent R. Johnson, and Chris D. Barton

Appendix S1

Table S1. Data used to estimate total annual contribution of leaf litter from ephemeral tributaries to Clemons Fork in Robinson Forest, KY.

Parameter	Value	References
Total ephemeral channel length (L)	52.64 km	Fritz et al. 2013
Ephemeral channel width (W)	0.57 m	This study
Total ephemeral channel area = (L x W)/2	15002.4 m^2	
Annual litterfall input	283 g AFDM m^{-2} y^{-1}<br>419 g AFDM m^{-2} y^{-1}<br>309 g AFDM m^{-2} y^{-1}<br>347 g AFDM m^{-2} y^{-1}<br>468 g AFDM m^{-2} y^{-1}	Newman et al. 2006<br><br>Littlefield et al. 2013
Lateral input = 25% of litterfall input	70.8 g AFDM m^{-2} y^{-1}<br>104.8 g AFDM m^{-2} y^{-1}<br>77.2 g AFDM m^{-2} y^{-1}<br>86.8 g AFDM m^{-2} y^{-1}<br>117.0 g AFDM m^{-2} y^{-1}	Benfield 1997
Total input and assume 15% leach loss (standing crop)	300.7 g AFDM m^{-2} y^{-1}<br>445.2 g AFDM m^{-2} y^{-1}<br>328.3 g AFDM m^{-2} y^{-1}<br>368.7 g AFDM m^{-2} y^{-1}<br>497.2 g AFDM m^{-2} y^{-1}	Richardson 2000
Total ephemeral channel area leaf mass	4511.03 kg AFDM y^{-1}<br>6678.88 kg AFDM y^{-1}<br>4925.48 kg AFDM y^{-1}<br>5531.20 kg AFDM y^{-1}<br>7459.94 kg AFDM y^{-1}	
Mean (range) proportion of leaves exported per day from ephemeral channels	Nov-Dec: 0.0316 (0.0238 – 0.0358)<br>Dec-Jan: 0.0309 (0.0246 – 0.0357)<br>Jan-Feb: 0.0250 (0.0177 – 0.0275)<br>Feb-Mar: 0.0313 (0.0236 – 0.0356)<br>Mar-Apr: 0.0359 (0.0290 – 0.0382)<br>Apr-May: 0.0204 (0.0080 – 0.0280)	This study
Mean (range) total annual leaf mass exported downstream	5821.21 kg AFDM y^{-1} (4510.54 – 7459.94)	
Mean (range) annual leaf mass exported per kilometer of ephemeral channel	110.58 kg AFDM km^{-1} y^{-1} (85.69 – 141.72)	
